# Absence of *optineurin* (*OPTN*) gene mutations in Taiwanese patients with juvenile-onset open-angle glaucoma

**Published:** 2008-03-11

**Authors:** Yung-Chang Yen, Jiann-Jou Yang, Ming-Chih Chou, Shuan-Yow Li

**Affiliations:** 1 Institute of Medicine, Chung Shan Medical University, Taichung, Taiwan; 2 Genetics Laboratory, Department of Biomedical Sciences, Chung Shan Medical University, Taichung, Taiwan; 3 Department of Ophthalmology, Chi Mei Medical Center Liouying, Tainan, Taiwan

## Abstract

**Purpose:**

To investigate sequence variants in the *optineurin* (*OPTN*) gene in patients with juvenile–onset open-angle glaucoma (JOAG) in Taiwan.

**Methods:**

We analyzed the sequence variants of *OPTN* in 51 unrelated Taiwanese probands with JOAG and in 51 control group subjects who did not have JOAG. Genomic DNA was extracted from the individuals and subjected to polymerase chain reaction (PCR) to amplify all 16 exons and flanking introns of *OPTN*. The amplified products were then screened for base variants by autosequence. Data from the two study groups were then compared using Fisher’s exact test and Armitage's trend test.

**Results:**

Fifteen variants of *OPTN* were found in the 51 JOAG patients and 51 unrelated normal controls. Two were missense variants (M98K and K322E), one was a synonymous codon change (T34T), and 12 were changes in the noncoding sequences. Seven of the variants have been reported and eight were novel. All of the sequence changes were found in patients with JOAG and in the normal controls except for variant c.-233+25C>G, which was found only in the control group. Allelic frequencies of these sequence changes did not differ significantly between patients and controls (p>0.05) except for the variant c.-233+25C>G (p<0.001). Genotype frequencies of c.-233+25C>G was shown to be significant between the two groups using Fisher’s two-tailed exact test (p<0.001) and Armitage's trend test (p=6.815e^−06^).

**Conclusions:**

Our data indicate that none of the mutations in *OPTN* are associated with JOAG. The variant M98K is not a risk factor and the variant c.-233+25C>G may be protective against glaucoma in Taiwanese.

## Introduction

Glaucoma is a heterogeneous group of optic neuropathies that lead to optic nerve atrophy and a permanent loss of vision [1]. Glaucoma is the second leading cause of blindness worldwide with a prevalence of 0.15% in the total population and approximately 2%–4% in individuals over the age of 40 years [2]. Glaucoma is also a multifarious disease reportedly caused by the interaction of several environmental and genetic factors [3]. The most frequent form of glaucoma is primary open-angle glaucoma (POAG). POAG is a complex disease of unknown etiology that leads to a progressive loss of the visual field. POAG has been subdivided into two groups according to the age at onset. Adult-onset open-angle glaucoma is a condition typically diagnosed after the age of 40 years; juvenile-onset open-angle glaucoma (JOAG) is a disease most often diagnosed in patients younger than 35 years of age [4]. The definition of juvenile glaucoma is not consistent in the literature. The condition is variously described as a subset of infantile glaucoma and as an early onset form of chronic open-angle glaucoma. Whether this disease actually represents a distinct clinical entity has also been questioned by several researchers [5].

At least 14 gene loci (GLC1A-N) have been linked to POAG (OMIM 137760). Among these loci, three genes (*MYOC* encoding myocilin, *OPTN* encoding optineurin, and the *WDR36* encoding T-cell activation WD repeat-containing protein [TAWDRP] gene) have been considered to harbor the mutations that result in POAG [6–12].

Primary open angle glaucoma-1E (GLC1E) is caused by mutation in the *OPTN* gene (OMIM 602432). The *OPTN* gene locus was mapped to chromosome 10p15-p14 by linkage analysis of the gene in a large British family with a classic form of normal tension glaucoma [9]. The *OPTN* gene consists of 16 exons; the first three are noncoding exons in the 5′ untranslated region followed by 13 exons that code for a 577 amino acid protein. *OPTN* is expressed in ocular tissues such as the retina, trabecular meshwork, and nonpigmented ciliary epithelium [13]. Mutations in *OPTN*, arguably the second POAG gene, were initially found in 16.7% of families with hereditary and adult-onset POAG and in 12% of sporadic patients with POAG. The majority of them had an intraocular pressure (IOP) of less than 22 mmHg [13]. Mutations in *OPTN* were also found to account for 1.6% of sporadic POAG in Chinese patients [14].

Previous studies in Western populations show a significant racial/ethnic variation in glaucoma prevalence between white people and black people, largely related to variation in the prevalence of primary open-angle glaucoma (POAG) [15–17]. Until recently, similar data have been described in Asian populations [18]. Based on the survey of low vision (WHO criteria), the prevalence of primary open-angle glaucoma in the elderly Chinese was about 2% in Taiwan [19]. In another study, glaucoma was the leading cause of low vision and blindness in Taiwan [20]. However, the gene study of glaucoma just begin to be investigated [21] and the contribution of variants in *OPTN* for JOAG in Taiwan is currently unknown. In this study, we aimed to determine the variants of the 16 exons and flanking introns of *OPTN* using comparative genetic analysis between genomic DNA from unrelated normal individuals and JOAG patients in Taiwan.

## Methods

### Subject selection

A total of 1,210 patients who presented to the Department of Ophthalmology at the Kuo General Hospital with symptoms of glaucoma were evaluated. All study patients received more than two complete ocular examinations, each comprising slit-lamp testing, IOP measurement, a fundus examination, and a visual-field examination. Patients were defined as having JOAG if they were first diagnosed before the age of 35 years and found to have an intraocular pressure (IOP) greater than 22 mmHg, a cup/disc ratio greater than 0.5 or one with an asymmetric appearance, a visual-field loss characteristic of glaucomatous change, and an open-angle width ranging from Shaffer grade II to IV without any other apparent secondary cause (e.g., traumatically or surgically induced). Of the 1,210 individuals screened, JOAG was diagnosed in 51 unrelated patients. These patients were included in the patient group for subsequent *OPTN* genetic analysis. Most of these subjects have been reported in a previous study of *MYOC* mutations in Taiwanese patients with JOAG [20]. Subjects with mutations in *MYOC* were also included in the study.

Fifty-one randomly selected normal individuals over 50 years of age were included as the control group. None of them featured JOAG. Individuals in the control group also received complete ocular examinations as described above to exclude the possibility of glaucoma. The study protocol was approved by the Institutional Review Board of the Kuo General Hospital and was performed in accordance with the World Medical Association's Declaration of Helsinki (2000). All of the participants provided signed informed consent to participate in the study after details of the study were thoroughly explained to them.

### Detection of mutations in *OPTN*

DNA samples were collected from 10 ml of peripheral blood acquired from each of the 102 individuals and purified using a Gentra DNA Blood Kit (Gentra Systems Inc., Minneapolis, MN) according to the manufacturer’s directions. The quality and quantity of purified genomic DNA were determined by gel electrophoresis and spectrophotometry, respectively. Mutations in the 16 exons and flanking introns of *OPTN* were screened by direct sequencing (GenBank NT_077569). The intragenic primers used for polymerase chain reaction (PCR) are listed in Table 1. In brief, PCR was performed in a reaction volume of 25 μl containing 100 ng of genomic DNA, 200 μM dNTP, 0.25 units of proTaq DNA polymerase (Promega Corporation, Madison, WI), and 200 μM of intragenic primers. The PCR products were purified using a PCR Purification Kit (QIAGEN GmbH, Hilden, Germany) and then subjected to PCR-directed DNA sequencing using a DNA Sequencing Kit (Applied Biosystems Corporation, Foster City, CA). Sequencing was performed on an Applied Biosystems model 377 automated sequencer (Applied Biosystems Corporation). Sequence data were compared with the published sequence of *OPTN* (GenBank NM_001008211 and NT_077569).

**Table 1:** Oligonucleotide primer pairs for polymerase chain reaction.

**Name**	**Oligonucleotide**	**Annealing temperature (°C)**	**Region**	**Product size (bp)**
1F	AGGGAGCGGCTGGCTGTC	63.5+DMSO	Exon 1	302
1R	GGCGGGTACCGTTTTCAGG			
2F	TCCACATGGATGCCTCTACA	60	Exon 2	459
2R	TTCCCATGCAAATCTTCAAA			
3F	CTGGGATTACAGGCGTGAG	54	Exon 3	628
3R	GCCTTGCCAAATGCTAAATC		Exon 4	
5F	GGCTAAGCATGGCATCTTTC	64	Exon 5	460
5R	CTTCCAAGACCAGGCAAAAC			
6F	CTCAAAATCCTGGCCTCAAG	64+DMSO	Exon 6	575
6R	TTCAATTTGCCAGGACATGA			
7F	ATGGCGAGATGAAACTGACC	62.2	Exon 7	404
7R	ATTTGACCTCCGGTGACAAG			
8F	TTGGAGAATGTTCTGGAAAGC	60.9	Exon 8	485
8R	CAGAAAGCACATTGCTTGGA			
9F	TTTGAAAACCCCTGATCCTTT	62.2	Exon 9	496
9R	TTGCAGTGAGCTGAGATCGT			
10F	GGATTGATTCACCAGCCAGT	62.2	Exon 10	449
10R	AAGTTCTCCAGTCCCCAACC			
11F	CCTTGGGGTTTGTTTAAAAGC	62.2	Exon 11	489
11R	TCACCCCACGACCTACTAGC			
12F	GCTAGTAGGTCGTGGGGTGA	64	Exon 12	420
12R	CGCAATAAAGCACATTACACA			
13F	TATGTTGCCCAGGCTTGTCT	65+DMSO	Exon 13	518
13R	AGATCCACTGAGCACTTTCCA			
14F	TGCTATCGGAATGTACCTGGA	60	Exon 14	506
14R	TCACATGAAGTGTAAGTGAAGCAA			
15F	TGAACCTTGGCAGTGTAGTTTG	64	Exon 15	373
15R	GATTCGGTGGGTAATGGATG			
16F	CCTGTGCTCATGTCCCACTA	59+DMSO	Exon 16	430
16R	CTCCCAAAGTGCTGGGATTA			

Primers were used in the amplification of *OPTN* exons and were used in sequencing reactions. Primers located in introns were placed far enough away from the exon boundaries to allow visualization of the sequence of the splice site. In the table, “F” indicates the forward strand and “R” indicates the reverse strand. In additional, bp represents base pair and DMSO represents dimethylsulfoxide in this table.

### Statistical analysis

Significant differences in allele frequencies between JOAG patients and controls were determined by the Fisher’s two-tailed exact test. The genotype frequencies between JOAG patients and controls were determined by Fisher’s two-tailed exact test and Armitage's trend test. A p value less than 0.05 represented a statistically significant difference between JOAG patients and control individuals. Variants were tested for Hard-Weinberg Equilibrium (HWE) using the normal 2×3 Χ^2^ 1 degree of freedom (df) test of goodness-of-fit to HW proportions.

## Results

This study surveyed mutations in all 16 exons of *OPTN* including the flanking intronic sequences and coding region in 102 Taiwanese individuals. The patient group comprised 51 unrelated patients with JOAG (30 males and 21 females). The patients ranged in age from 17 to 61 years at the time of the study. There were 51 unrelated individuals (28 males and 23 females) without any evidence of glaucoma in the control group. The clinical features of the patients and control subjects are listed in Table 2.

**Table 2:** Clinical characteristics of participants.

**Variable**	**JOAG (n=51)**	**Control (n=51)**
Age at the time of the study (mean ± SD)	35.8 ± 10.1	70.4 ± 9.9
Female (%)	41.2 (21/51)	45.1 (23/51)
Male (%)	58.8 (30/51)	54.9 (28/51)
IOP OD (mean ± SD)	18.45 ± 4.16	11.94 ± 2.75
IOP OS (mean ± SD)	19.08 ± 4.22	11.86 ± 3.09
C/D OD (mean ± SD)	0.74 ± 0.14	0.42 ± 0.08
C/D OS (mean ± SD)	0.73 ± 0.15	0.42 ± 0.08

Clinical characteristics of 102 Taiwanese individuals including 51 JOAG patients under topical anti-glaucoma agent medication and 51 unrelated normal controls are listed in the table. IOP indicates intraocular pressure; C/D indicates cup-disc ratio of the optic nerve; OD indicates right eye; OS indicates left eye; and SD indicates standard deviation.

Fifteen sequence changes of *OPTN* were found in the 51 JOAG patients and 51 unrelated normal controls (Table 3). Two were missense changes, one was a synonymous codon change, and 12 were changes in the noncoding sequence. Apart from T34T, M98K, c.553–5C>T, c.553–10G>A, c.626+24G>A, c.779+20G>A, and c.1979–48C>A, the other eight sequence changes (c.-284G>A, c.-105G.>A, c.964A>G, c.-233+25C>G, c.166+66A>G, c.708–53T>C, c.882+109A>G, and c.1978+101A>C) were novel (Table 3). All of the sequence changes were found in the patients with JOAG and in the normal controls except for variant c.-233+25C>G, which was found only in the control group. However, the homozygous c.-105G>A variant was observed in three of the JOAG patients. In contrast, the homozygous c.-105G>A variant was found only in the normal control individuals (Table 4. M98K was identified in 17 patients with JOAG and in 14 normal control individuals. Five of the patients and two of the control individuals were homozygous for M98K (Table 4. In addition, the c.A964A<G (K322E) variant of *OPTN* was found in all of the patients with JOAG and in all of the normal controls in our study. All variants were found to be consistent with Hardy–Weinberg equilibrium expectations.

**Table 3:** Variants of the *OPTN* gene in this study.

**Genotype variants**	**Amino acid variants**	**Location**	**Predicted effect**	**Reference**
c.-284G>A	N	Exon 1	—	This study
c.-105G.>A	N	Exon 2	—	This study
c.102G>A	T34T	Exon 4	synonymous change	[13]
c.293T>A	M98K	Exon 5	missense change	[13]
c.964A>G	K322E	Exon 10	missense change	This study
c.-233+25C>G	N	Intron 1	—	This study
c.166+66A>G	N	Intron 4	—	This study
c.553–5C>T	N	Intron 6	—	[14,31]
c.553–10G>A	N	Intron 6	—	[14]
c.626+24G>A	N	Intron 7	—	[14]
c.708–53T>C	N	Intron 7	—	This study
c.779+20G>A	N	Intron 8	—	[14]
c.882+109A>G	N	Intron 9	—	This study
c.1978+101A>C	N	Intron 15	—	This study
c.1979–48C>A	N	Intron 15	—	[14]

Fifteen variants of *OPTN* were determined in the 51 JOAG patients and 51 unrelated normal controls. Two were missense variants (M98K and K322E), one was a synonymous codon change (T34T), and 12 were changes in the noncoding sequences. In addition, seven of the variants have been reported that it was indicated in reference position and eight were novel. The “N” indicates that the variant had no change in amino acid. The “dash” indicates that the variant had no determined effect.

**Table 4:** Genotype frequencies of *OPTN* variants in JOAG patients and normal control individuals.

**Genotype variants**	**Allele 1/ Allele 2**	**JOAG, % (n=51)**	**Control, % (n=51)**	p-Value*	**p-Value#**
c.-284G>A	G/G	66.7 (34/51)	70.6 (36/51)		
	G/A	13.7 (7/51)	25.5 (13/51)	0.023	0.153
	A/A	19.6 (10/51)	3.9 (2/51)		
c.-233+25C>G	C/C	100 (51/51)	62.7 (32/51)		
	C/G	0 (0/51)	25.5 (13/51)	<0.001	6.815e-06
	G/G	0 (0/51)	11.8 (6/51)		
c.-105G>A	G/G	84.3 (43/51)	92.2 (47/51)		
	G/A	9.8 (5/51)	7.8 (4/51)	0.299	0.106
	A/A	5.9 (3/51)	0 (0/51)		
c.102G>A	G/G	64.7 (33/51)	68.6 (35/51)		
	G/A	33.3 (17/51)	23.6 (12/51)	0.253	0.864
	A/A	2.0 (1/51)	7.8 (4/51)		
c.166+66A>G	A/A	64.7 (33/51)	68.6 (35/51)		
	A/G	33.3 (17/51)	23.5 (12/51)	0.253	0.864
	G/G	2.0 (1/51)	7.8 (4/51)		
c.293T>A	T/T	66.7 (34/51)	72.6 (37/51)		
	T/A	23.5 (12/51)	23.5 (12/51)	0.571	0.329
	A/A	9.8 (5/51)	3.9 (2/51)		
c.553–5C>T	C/C	2.0 (1/51)	0 (0/51)		
	C/T	31.3 (16/51)	41.2 (21/51)	0.410	0.557
	T/T	66.7 (34/51)	58.8 (30/51)		
c.553–10G>A	G/G	92.1 (47/51)	78.5 (40/51)		
	G/A	5.9 (3/51)	17.6 (9/51)	0.141	0.079
	A/A	2.0 (1/51)	3.9 (2/51)		
c.626+24G>A	G/G	96.1 (49/51)	84.3 (43/51)		
	G/A	3.9 (2/51)	15.7 (8/51)	0.092	0.050
	A/A	0 (0/51)	0 (0/51)		
c.779+20G>A	G/G	84.3 (43/51)	86.3 (44/51)		
	G/A	15.7 (8/51)	13.7 (7/51)	>0.999	0.779
	A/A	0 (0/51)	0 (0/51)		
c.882+109A>G	A/A	52.9 (27/51)	35.3(18/51)		
	A/G	35.3 (18/51)	43.1 (22/51)	0.164	0.057
	G/G	11.8 (6/51)	21.6 (11/51)		
c.708–53T>C	T/T	92.2 (47/51)	86.2 (44/51)		
	T/C	3.9 (2/51)	2.0 (1/51)	0.446	0.212
	C/C	3.9 (2/51)	11.8 (6/51)		
c.964A>G	A/A	0 (0/51)	0 (0/51)		
	A/G	0 (0/51)	0 (0/51)	1.000	NA
	G/G	100 (51/51)	100 (51/51)		
c.1978+101A>C	A/A	47.1 (24/51)	54.9 (28/51)		
	A/C	47.1 (24/51)	37.3 (19/51)	0.669	0.632
	C/C	5.8 (3/51)	7.8 (4/51)		
c.1979–48C>A	C/C	62.7 (32/51)	58.8 (30/51)		
	C/A	25.5 (13/51)	27.5 (14/51)	0.920	0.676
	A/A	11.8 (6/51)	13.7 (7/51)		

Fisher’s two-tailed exact test (p Value*) and Armitage's trend test *(*p Value^#^**)** were used to compare the genotype frequencies of various sequence changes between JOAG and control subjects. Genotype frequency of c.-284G>A variant was shown to be significant between two groups using Fisher’s two-tailed exact test (p=0.023; p<0.05). However, we used Armitage's trend test to compare the genotypic frequency of the c.-284G>A variant was not significant (p=0.153). In addition, the c.-233+25C>G genotype frequency was shown to be significant between two groups using Fisher’s two-tailed exact test (p<0.001) and Armitage's trend test (p=6.815e-06). Apart from c.-284G>A and c.-233+25C>G, the other 13 variants did not differ significantly between patients and controls (p>0.05).

To understand the distribution of *OPTN* sequence changes in JOAG patients and normal control individuals, we used Fisher’s two-tailed exact test to compare the allelic and genotypic frequencies of the sequence changes between the two groups. Genotype frequencies were consistent with HWE proportions for all variants. We found that the allelic frequencies of these sequence changes did not differ significantly between patients and controls (p>0.05) except for variant c.-233+25C>G (p<0.001; Table 5. However, we observed that the c.-284G>A variant (p=0.023) and the c.-233+25C>G variant (p<0.001) of *OPTN* was associated with JOAG whereas the other variants of *OPTN* were not associated with JOAG in genotypic frequency analysis using Fisher’s two-tailed exact test (p>0.05; Table 4. Further, we used Armitage's trend test to compare the genotypic frequencies of the c.-284G>A and c.-233+25C>G variants between the two groups. The variant c.-233+25C>G was significant between patients and controls (p=6.815e^−06^), but variant c.-284G>A was not significant (p=0.153). In additional, we included another 51 control individuals for the c.-284G>A variant. We found the c.-284G>A variant in 30 normal control individuals, including five (4.9%; 5/102) who were homozygous and 25 (24.5%; 25/102) who were heterozygous. These results, the allelic and genotypic frequencies of the c.-284G>A variant between the two groups, are similar with the 51 normal control individuals (data not shown). Taken together, we suggested that the c.-284G>A variant may not be a risk factor for JOAG and the c.-233+25C>G variant may be protective against glaucoma in the Taiwanese.

**Table 5:** Allele frequencies of *OPTN* variants in this study.

**Variants**	**Allele Frequency (%)**			**Genotype**	
Genotype	JOAG (n=102)	Control (n=1–2)	p-value	JOAG (n=51)	Control (n=51)
c.-284G>A	G: 0.74	G: 0.83	0.089	10/7/1934	2/13/1936
	A: 0.26	A: 0.17			
c.-233+25C>G	C: 1.00	C: 0.75	<0.001	0/0/51	6/13/1932
	G: 0.00	G: 0.25			
c.-105G.>A	G: 0.83	G: 0.96	0.06	3/5/1943	0/4/47
	A: 0.17	A: 0.04			
c.102G>A	G: 0.81	G: 0.80	0.859	1/17/1933	4/12/1935
	A: 0.19	A: 0.20			
c. IVS4+66A>G	A: 0.81	A: 0.80	0.859	1/17/1933	4/12/1935
	G: 0.19	G: 0.20			
c.293T>A	T: 0.78	T: 0.84	0.281	5/12/1934	2/12/1937
	A: 0.22	A: 0.16			
c.553–5C>T	C: 0.18	C: 0.21	0.254	34/16/1	30/21/0
	T: 0.72	T: 0.79			
c.553–10G>A	G: 0.95	G:0.87	0.048	1/3/1947	2/9/1940
	A: 0.05	A:0.13			
c.626+24G>A	G: 0.98	G:0.95	0.618	0/2/49	0/8/43
	A: 0.02	A:0.05			
c.779+20G>A	G: 0.92	G:0.93	0.249	0/8/43	0/7/44
	A: 0.08	A:0.07			
c.882+109A>G	A: 0.53	A:0.57	0.789	6/18/2027	11/22/2018
	G: 0.47	G:0.43			
c.708–53T>C	T: 0.94	T:0.87	0.574	2/2/1947	6/1/1944
	C: 0.06	C:0.13			
c.964A>G	A: 0.00	A:0.00	1	51/0/0	51/0/0
	G: 1.00	G:1.00			
c.1978+101A>C	A: 0.71	A:0.74	0.64	3/24/2024	4/19/2028
	C: 0.29	C:0.26			
c.1979–48C>A	C: 0.75	C:0.73	0.632	6/13/1932	7/14/1930
	A: 0.25	A:0.27			

All subjects were screened and scored for each variant listed. Fisher’s two-tailed exact test was used to compare the allele frequencies of various sequence changes between JOAG and control subjects. The genotype numbers indicate homozygote variant/heterozygote variant/no variant.

## Discussion

Information regarding the role that *MYOC* and *OPTN* play in the development of JOAG in Taiwanese is insufficient. In our previous study, we identified four *MYOC* mutations and six polymorphisms in Taiwanese patients with JOAG. The prevalence of *MYOC* gene mutations was 12.5% [21]. However, the contribution of *OPTN* sequence variations to JOAG in Taiwan has not been analyzed. In this study, we found 15 variants of *OPTN* in the 51 JOAG patients and in the 51 unrelated normal controls. Two were missense variants (M98K and K322E), one was a synonymous codon change (T34T), and 12 were changes in the noncoding sequences.

In previous studies, at least seven mutations of *OPTN* have been reported to be associated with adult-onset POAG [13,14,22]. Of the seven *OPTN* mutations that have been identified in patients with adult-onset POAG, one is located in exon 4 (E50K) [13,21], one is located in exon 5 (E103D) [14], two are located in exon 6 (c.691_692insAG and V148V) [13,14,22], one is located in exon 14 (H486R) [14], one is located in exon 16 (R545Q) [13,22], and one is located in intron 13 (IVS 13+21C>G) [14]. Although none of the aforementioned mutations were detected in this study, we identified 15 sequence variants of *OPTN*. These sequence changes are not classified as causative mutations but as polymorphisms because the frequencies were similar between the patient and normal control groups.

Previous reports have indicated that mutations of *OPTN* are responsible for moderate to mild forms of late onset glaucoma and are specific for normal tension glaucoma (NTG) [13]. *OPTN* mutations have been found in 16.7% of patients with the hereditary form of NTG and in about 1%–2% of patients with sporadic POAG [13,14,23]. However, two subsequent studies on Caucasian POAG patients, one study involving 801 patients of variable age onset [23] and one involving 86 adult-onset patients [24], found no glaucoma-causing mutations in *OPTN*. In Japan, 148 patients with NTG and 165 with hypertension glaucoma had no specific glaucoma-causing mutations in *OPTN* [25]. In addition, a report also indicated that no mutation of *OPTN* was associated with JOAG patients in the Philippines [26]. Our results are consistent with those reported by Wang et al. [26] who found that *OPTN* mutations were absent in JOAG patients. Therefore, we suggest that *OPTN* mutations are not related to the development of JOAG in Taiwanese.

The M98K variant of OPTN was reported to be associated with POAG in patients with normal or elevated IOP. The allele frequency of the M98K variant was 13.6% in POAG patients and only 2.1% in the control population. The result indicated that M98K was very strongly associated with glaucoma [13]. In our study, M98K was found in 33.3% of JOAG patients (17/51) and 27.5% of individuals in the control group (14/51); however, the frequency of the M98K variant did not differ significantly between patients and controls (p=0.571). In fact, investigations of the OPTN M98K variant in patients with POAG have yielded conflicting findings so far. Recently, a meta-analysis of all published works to date on the OPTN M98K variant in glaucoma patients did not show a strong role for the OPTN M98K variant in glaucoma, autosomal dominant optic atrophy (ADOA), or Leber hereditary optic neuropathy (LHON) [27]. In addition, a large study that investigated the allele frequencies of the M98K variant in *OPTN* also found no significant differences in allele frequencies between POAG patients and controls in Asian, African, Hispanic, or Caucasian populations [22]. However, some studies have indicated that the M98K variant is associated with POAG within populations in Japan [22,28] and Europe [29]. Thus, Ayala-Lugo et al. [22] suggested that ancestry is a significant confounding variable. In addition, Melki et al. [30] reported that M98K was associated with decreased initial intraocular pressure (IOP) in French patients but was not significantly associated with decreased IOP in Moroccan patients. Therefore, we believe that the association between the M98K variant and glaucoma can be attributed to racial variation. Based on our results and those from previous studies, the M98K variant of *OPTN* is not a risk factor associated with JOAG in Taiwan.

In our study, the c.-233+25C>G variant was identified in the 19 normal control individuals but was not found in the JOAG individuals. Thirteen of the control individuals (25.5%; 13/51) were homozygous, and six of the control individuals (11.8%; 6/51) were heterozygous for c.-233+25C>G. The c.-233+25C>G allele frequencies and genotype frequencies were shown to be significant between two groups using Fisher’s two-tailed exact test (p<0.001) and Armitage's trend test (p=6.815e^−06^; shown in Table 4 and Table 5). Therefore, we suggest that the c.-233+25C>G variant may be protective against glaucoma in the Taiwanese. Further research into this association would be interesting. Expression studies and functional assays may be needed to determine whether these sequence variants play a role as transcription activators or inhibitors. This might ultimately shed light on the mechanisms relevant to the development of glaucoma. In addition, A964 nucleotide of *OPTN* has been reported in GenBank NM_001008211. In contrast, the G964 nucleotides were found in all patients with JOAG and in all normal controls in our study. One possible explanation for such a difference in nucleotides is attributed to racial variations. Therefore, the c.964A>G variant is not associated with JOAG.
